# Transcriptomal profiling of the cellular response to DNA damage mediated by Slug (Snai2)

**DOI:** 10.1038/sj.bjc.6604084

**Published:** 2008-01-08

**Authors:** M Pérez-Caro, C Bermejo-Rodríguez, I González-Herrero, M Sánchez-Beato, M A Piris, I Sánchez-García

**Affiliations:** 1 Experimental Therapeutics and Translational Oncology Program, Instituto de Biología Molecular y Celular del Cáncer (IBMCC), CSIC/Universidad de Salamanca, Salamanca, Spain; 2 Molecular Pathology Program, Centro Nacional de Investigaciones Oncologicas, Madrid, Spain

**Keywords:** Snai2, MEFs, microarray analysis, stem cells, DNA damage

## Abstract

Snai2-deficient cells are radiosensitive to DNA damage. The function of *Snai2* in response to DNA damage seems to be critical for its function in normal development and cancer. Here, we applied a functional genomics approach that combined gene-expression profiling and computational molecular network analysis to obtain global dissection of the Snai2-dependent transcriptional response to DNA damage in primary mouse embryonic fibroblasts (MEFs), which undergo p53-dependent growth arrest in response to DNA damage. Although examination of the response showed that overall expression of p53 target gene expression patterns was similarly altered in both control and Snai2-deficient cells, we have identified and validated candidate Snai2 target genes linked to *Snai2* gene function in response to DNA damage. This work defines for the first time the effect of Snai2 on p53 target genes in cells undergoing growth arrest, elucidates the Snai2-dependent molecular network induced by DNA damage, points to novel putative Snai2 targets, and suggest a mechanistic model, which has implications for cancer management.

Uncommitted progenitor cells express Snai2 and aberrant activation of Snai2 pathways is key in the development of cancers derived from many tissues (Inoue *et al*, 2002; Perez-Losada *et al*, 2002; Perez-Mancera *et al*, 2005). The implication of *SNAI2* in human cancer seems to be wider than initially expected (Elloul *et al*, 2005; Gupta *et al*, 2005; Shih *et al*, 2005; Bermejo-Rodriguez *et al*, 2006; Come *et al*, 2006). Nevertheless, the molecular mechanisms by which *SNAI2* participates in these biological processes are not yet clear. *In vitro* studies have shown that Snai2 confers resistance to cell death induced by the withdrawal of survival factors (Inoue *et al*, 2002; Perez-Losada *et al*, 2002; Perez-Mancera *et al*, 2005). To understand the relevance of SNAI2 to human cancer Snai2-expressing mice were generated (Perez-Mancera *et al*, 2005). The analysis of the Snai2-expressing mice identified that ‘uncontrolled’ Snai2 expression induces cancer in mice. These findings further indicated that overexpression of SNAI2 by human tumours could be of importance to both cell fate selection by genotoxic anticancer agents (Perez-Mancera *et al*, 2005) and clinical management of human cancer patients (Elloul *et al*, 2005; Shih *et al*, 2005; Bermejo-Rodriguez *et al*, 2006; Come *et al*, 2006). These results suggested that Snai2 expression was protecting cells from death by genetic alterations as a consequence of an inherent, basal level of genetic instability (Sanchez-Garcia, 1997; Pérez-Caro *et al*, 2005; Perez-Mancera *et al*, 2005). Consistent with this interpretation, in the haematopoietic system Snai2-deficient cells are radiosensitive to DNA damage (Inoue *et al*, 2002; Perez-Losada *et al*, 2003; Wu *et al*, 2005). In fact, *SNAI2* expression seems to be associated with a patient's resistance to chemotherapeutic agents in human mesotheliomas (Catalano *et al*, 2004). Thus, constitutive activation of Snai2 could confer resistance properties to the tumour-target cells connecting DNA damage with the requirement of a critical level of Snai2 for cancer development. In agreement with these results, recently it has been shown Snai2 is a p53 target that antagonises p53-mediated apoptosis of haematopoietic progenitors (Wu *et al*, 2005). However, the effect of Snai2 on p53 target genes remains an open question (Haupt *et al*, 2006).

In this study, we have investigated the role of *Snai2* in DNA damage. To explore the role of Snai2 in DNA damage response, we used primary mouse embryonic fibroblasts (MEFs), which undergo p53-dependent growth arrest in response to DNA damage. Snai2 wild-type and null MEFs were subjected to DNA damage and the global gene expression patterns were examined. Although overall the expression of the majority of the spotted genes was not altered in Snai2-deficient cells compared to control MEFs, we have identified candidate Snai2 target genes linked to *Snai2* gene function in response to DNA damage.

## MATERIALS AND METHODS

### Preparation of MEF RNA populations

Wild-type, p53−/− and Snai2-deficient MEFs were isolated as described previously (Bermejo-Rodriguez *et al*, 2006). To prepare the RNA population for microarray studies, wild-type and Snai2-deficient MEFs (passage 4) were synchronised by growing to confluence in DMEM plus 10% FCS for 3 days. To stimulate re-entry into the cell cycle, the cells were reseeded into DMEM plus 10% FCS at 1.0 × 10^6^ cells per 10-cm dish. After 6 h, 0.2 *μ*g ml^−1^ doxorubicin (Sigma-Aldrich, Madrid, Spain) was added to induce G1 arrest. After 12 h treatment, cells were collected by trypsinisation and frozen as a pellet for subsequent RNA and/or protein preparation. Dishes of both untreated and treated wild-type and Snai2-deficient MEFs were collected after 16 h of doxorubicin treatment for FACS analysis to verify a p53-dependent G1 arrest as described previously (Attardi *et al*, 2000).

*γ*-Irradiation was performed using a ^137^Cs source. Asynchronously growing wild-type, p53*−/−* and Snai2-deficient MEFs were treated with 5 and 8 Gray (Gy) of *γ*-irradiation, which induces a well-characterised arrest in both G1 and G2 (Kastan *et al*, 1992). We chose to examine the effects at 18 h, a time point at which these arrest responses have been demonstrated previously.

#### RNA extraction

Total RNA was isolated in two steps using TRIzol (Life Technologies Inc., Grand Island, NY, USA) followed by Rneasy Mini-Kit (Qiagen Inc., Valencia, CA, USA) purification following the manufacturer's RNA Clean-up protocol with the optional on-column DNase treatment. The integrity and the quality of RNA was verified by electrophoresis and its concentration measured.

### Microarray procedures

A measure of 30 *μ*g of total RNA from each sample was directly labelled with cyanine 3-conjugated dUTP (Cy3), whereas 30 *μ*g of RNA from the Universal Mouse Reference RNA (Stratagene, VWR International, Spain) was labelled with cyanine 5-conjugated dUTP (Cy5) as reference. For all of the microarray studies the CNIO MouseChip was used (Bermejo-Rodriguez *et al*, 2006). Hybridisations were performed as described (Bermejo-Rodriguez *et al*, 2006). After washing, the slides were scanned using a Scanarray 5000 XL (GSI Lumonics Kanata, Ontario, Canada) and images were analysed with the GenePix 4.0 program (Axon Instruments Inc., Union City, CA, USA). All experiments were repeated four times using cells from different embryos.

#### Data analysis

Data obtained from each hybridisation were stored in a database for analysis. The Cy3 : Cy5 ratios were normalised to the median ratio value of all of the spots in the array. After normalisation, spots with intensities for both channels (sum of medians) less than that of the local background were discarded. The ratios of the remaining spots were log transformed (base 2), and duplicated spots on the MouseChip were averaged to the median. To obtain the expression profile of wild-type and Snai2-deficient MEFs, we referred the ratios of control and Snai2-deficient DNA-damaged cells to the undamaged counterparts. The Ingenuity Pathways Analysis program (http://www.ingenuity.com/index.html) was used to further analyse the cellular functions and pathways that were significantly regulated by Snai2 in response to DNA damage.

#### Quantitative RT–PCR

Control and Snai2−/− MEF RNA (1 *μ*g) was reverse-transcribed by using Advantage RT for PCR kit (BD Biosciences, Becton Dickinson, Madrid, Spain). SYBR Green PCR Master mix (Applied Biosystems, USA) was used for template amplification with the primers specific for each of the transcripts examined. PCR with RT sample were used as negative controls. Thermocycling for all targets were carried out in 30 *μ*l reaction for 40 cycles in triplicate. Each cycle consisted of 94°C for 15 s, 56°C for 30 s and 72°C for 30 s. Incorporation of the SYBR Green dye into PCR products was monitored in real time with an ABI PRISM 7000 sequence detection system (Applied Biosystems). SDS system software was used to convert the fluorescent data into threshold cycle (*C*_t_) at which exponential amplification of products begins. The differences in the *C*_t_ values (d*C*_t_) between the transcript of interest and endogenous control (GAPDH) were used to determine the relative expression of the gene in each sample and the dd*C*_t_ method was used to calculate fold expression. To determine correlation between expression of two genes in the same set of samples d*C*_t_ values were used to calculate regression coefficient.

#### Western blot analysis

Western blot analysis of control, p53−/−and Snai2−/− MEFs were carried out essentially as described (Castellanos *et al*, 1997). Extracts were normalised for protein content by Bradford analysis (Bio-Rad Laboratories Inc., Melville, NY, USA) and Coomassie blue gel staining. Lysates were run on a 10% SDS–PAGE gel and transferred on to a PVDF membrane. After blocking, the membrane was probed with the following primary antibodies: p53 (Ab3, Oncogene Research), Phospho-p53 (Ser15) (no. 9284, Cell Signaling Technology, IZASA, Barcelona, Spain), Puma/bbc3 (P4743, Sigma), Bid (sc-11423, Santa Cruz Biotechnology, Quimigen, Madrid, Spain), p27 (sc-1641, Santa Cruz Biotechnology), Atm (sc-1214, Santa Cruz) and actin (I-19) (sc-1616, Santa Cruz Biotechnology). Reactive bands were detected with an ECL system (Amersham, GE Healthcare, Madrid, Spain).

## RESULTS

### Normal G1 arrest in Snai2-deficient MEFs in response to doxorubicin treatment

**Figure 1:**
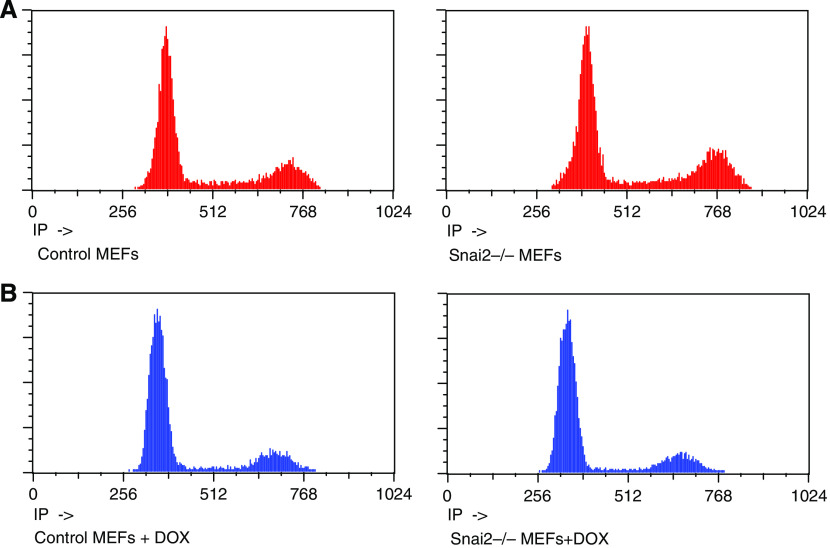
Doxorubicin induces a G1 arrest in Snai2-deficient mouse embryonic fibroblasts (MEFs). Dishes of both untreated (**A**) treated (**B**) wild-type and Snai2-deficient MEFs were collected after 16 h of doxorubicin treatment for FACS analysis to verify a G1 arrest. FACS profiles include G1-arrested without and with doxorubicin of both wild-type and Snai2-deficient MEFs.

Expression patterns were studied in wild-type MEFs and Snai2-deficient MEFs to search for Snai2-regulated species in response to DNA damage. We chose to use the DNA-damaging agent doxorubicin for this study as it effectively induced p53-dependent G1 arrest in MEFs (Attardi *et al*, 2000). To prepare RNA for screen wild-type MEFs and Snai2-deficient MEFs were treated with 0.2 *μ*g ml^−1^ doxorubicin for 12 h. This time point was chosen because it had previously shown it to be a point of peak expression of known p53 target genes (Attardi *et al*, 2000). That G1 arrest had occurred was confirmed by FACS analysis, which showed a G1 arrest in both the wild-type and the Snai2-deficient cells (Figure 1).

### Normal p53 activation in Snai2-deficient MEFs in response to doxorubicin treatment

**Figure 2:**
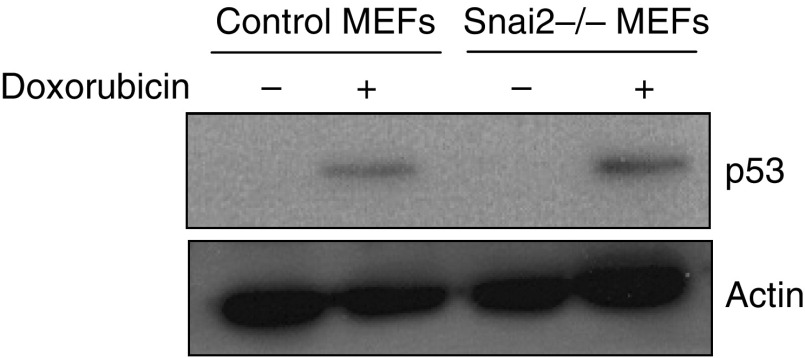
Levels of p53 protein in Snai2-deficient mouse embryonic fibroblast (MEF) cells in response to DNA damage treatment with doxorubicin. p53 protein was detected by western blotting in wild-type and Snai2-deficient MEFs. Actin was used as a loading control.

It has been previously shown that Snai2-deficient bone marrow cells are radiosensitive to DNA damage induced by *γ*-irradiation (Inoue *et al*, 2002; Perez-Losada *et al*, 2003; Wu *et al*, 2005). p53 is centrally involved in the cellular response to DNA damage (Attardi *et al*, 2000). Exposure to DNA damage agents causes an increase in the intracellular levels of p53 (Attardi *et al*, 2000). Thus, we next explored if the DNA damage-protective potential of Snai2 was based on interference with p53 activation in MEFs in response to doxorubicin treatment. As shown in Figure 2, we measured the p53 protein levels in wild-type MEFs and Snai2-deficient MEFs after DNA damage induced by doxorubicin. The activation of p53 in both control and Snai2-deficient cells was similar (Figure 2), indicating that p53 regulation in response to DNA damage is not affected in Slug-deficient MEF.

### Identification of Snai2-target genes in response to DNA damage by mouse cDNA Microarray analysis in MEFs

Expression analyses were performing with a mouse cDNA microarray (Mousechip-CNIO) containing 15 000 clones (Bermejo-Rodriguez *et al*, 2006). Expression patterns were studied in wild-type MEFs and Snai2-deficient MEFs. Three independent mouse cDNA microarrays were used to search for Snai2-regulated species in MEFs in response to DNA damage. To obtain a global view of the number of genes regulated by Snai2 in response to DNA damage, we hybridised differentially labelled RNA from control MEFs versus the Snai2-deficient MEFs to a mouse cDNA microarray. This system allows us to identify physiologically relevant Snai2 targets in response to DNA damage. Overall the expression of the majority of the spotted genes was not altered in Snai2-deficient cells in response to doxorubicin treatment.

**Figure 3:**
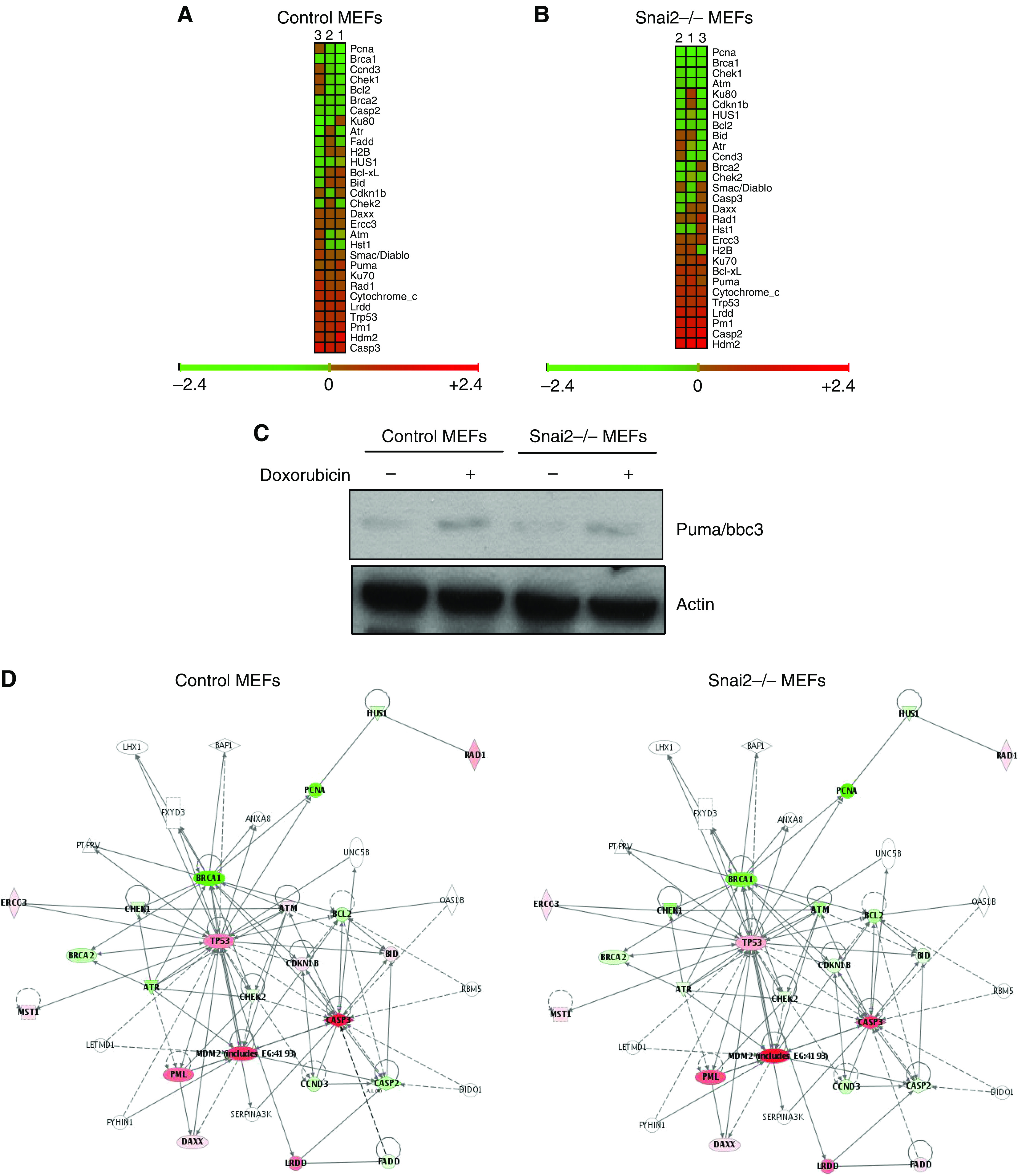

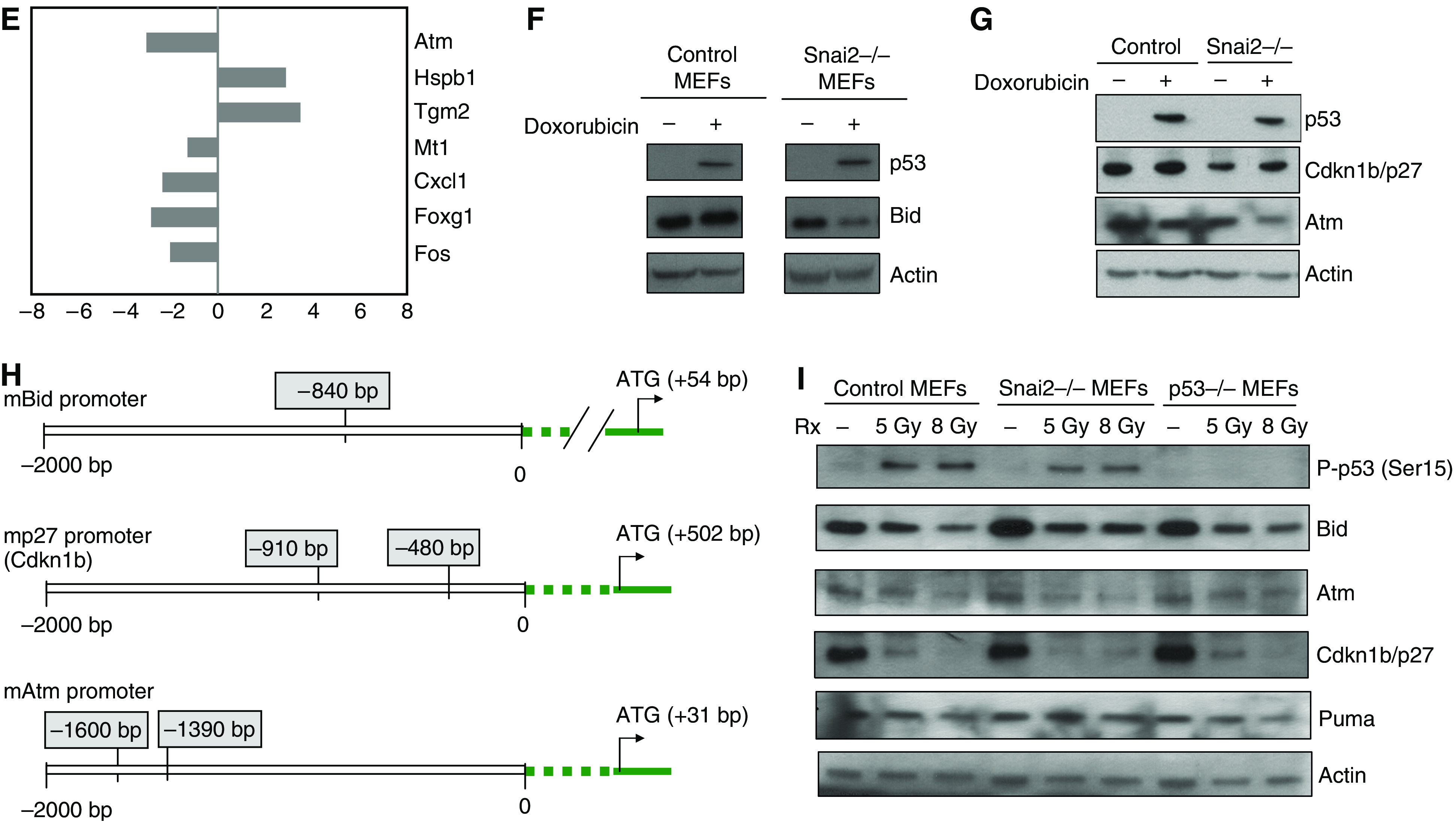
Graphical depiction of expression data for known target genes in response to DNA damage (cell cycle and apoptosis related genes) in wild-type mouse embryonic fibroblasts (MEFs) (**A**) and Snai2-deficient MEFs (**B**). Each gene (identified at right) represented by a single row of coloured boxes; each independent experiment is represented by one single column. (**C**) Puma expression in control MEFs and Snai2−/− MEFs in response to DNA damage treatment by doxorubicin. Puma protein analysis in control and Snai2−/− MEFs after DNA damage by western blot. Actin was used as a loading control. (**D**) Identification of Slug-dependent proteins using the Ingenuity database. Nodes are colour-coded in red (upregulated) or green (downregulated) according to their fold changes value. (**E**) Confirmation of the microarray results by qPCR performed as described in the Materials and Methods section. (**F** and **G**) Expression of Bid (**F**) and Atm and Cdkn1b/ p27 (**G**) in control MEFs and Snai2−/− MEFs in response to DNA damage treatment by doxorubicin. Bid, Atm and Cdkn1b/p27 protein analysis in control and Snai2−/− MEFs after DNA damage by western blot. Actin was used as a loading control. (**H**) Identification of Snai2 DNA-binding sites within promoter regions of murine *Atm*, *Bid* and *p27* genes (bp, base pairs). (**I**) Expression of p53, Puma, Bid, Atm and Cdkn1b/ p27 (**G**) in control, Snai2−/− and p53−/− MEFs in response to DNA damage treatment by *γ*-irradiation. p53 protein was detected by western blot in wild-type and Snai2-deficient MEFs. Puma, Bid, Atm and Cdkn1b/p27 protein analysis by western blot in control, Snai2−/− and p53−/− MEFs after DNA damage treatment with 5 and 8 Gy of *γ*-irradiation. Actin was used as a loading control.

To confirm that this microarray analysis could be used to identify Snai2 target genes, we first examined whether known target genes were modulated in control MEFs after doxorubicin treatment. As expected, the main sensor of DNA damage p53 was upregulated close to genes involved in cell cycle arrest (Ku70), apoptosis (Casp3, Puma, Lrdd) and DNA repair (mdm2, Rad1) (Figure 3A). Puma is a Bcl-2 family member involved in p53-induced apoptosis (Jeffers *et al*, 2003) and Puma/bbc3 expression was upregulated in control MEFs in response to DNA damage. On the contrary, survival gene expression was downregulated in control MEFs after DNA doxorubicin treatment (Figure 3A). Analysis of the expression of the same set of genes revealed that overall expression was not altered in Snai2-deficient cells in response to doxorubicin treatment (Figure 3B). The expression of the previously implicated Snai2-target gene Puma/bbc3 (Wu *et al*, 2005; Bermejo-Rodriguez *et al*, 2006) was not significantly modulated in Snai2-deficient cells in response to DNA damage. To confirm this result, we next measured the Puma/bbc3 protein levels in wild-type MEFs and Snai2-deficient MEFs after DNA damage induced by doxorubicin. In agreement with our previous observations (Bermejo-Rodriguez *et al*, 2006), the Puma/bbc3 protein levels in Snai2-deficient MEFs were lower than in control MEFs (Figure 3C). However, the activation of Puma/bbc3 in both control and Snai2-deficient cells in response to doxorubicin treatment was similar (Figure 3C), indicating that Puma/bbc3 regulation in response to DNA damage is not affected in Snai2-deficient MEF. These results suggest that p53 does not require Snai2 for Puma/bbc3 regulation in response to DNA damage in MEFs.

We further characterised our microarray data using the Ingenuity program (a software that identifies molecular networks by relating each gene entry with a database of known physical transcriptional or protein interactions). This study revealed the existence in the Snai2-dependent transcriptome of three proteins whose expression was altered in Snai2-deficient cells in response to DNA damage: Atm, Cdkn1b/p27 and Bid (Figure 3D). These results led us to demonstrate that these transcriptional factors were indeed downregulated in Snai2-deficient cells in response to DNA damage, by quantitative RT–PCR (qRT–PCR) in the case of Atm (Figure 3E) and by using specific antibodies to Bid, Atm and Cdkn1b/p27 (Figure 3F and G). Because Snai2 DNA-binding sites (Inukai *et al*, 1999) were present in promoter regions of *Atm,* and *Cdkn1b/p27* and *Bid* (Figure 3H), Snai2 might be directly involved in the control of transcription-repression of these targets.

We next examined if Snai2 was regulating these three targets, *Atm*, *Cdkn1b/p27* and *Bid*, in response to DNA damage by *γ*-irradiation. To prepare protein for analysis, wild-type MEFs, p53−/− and Snai2-deficient MEFs were treated with 5 and 8 Gy of *γ*-irradiation, which induces a well-characterised arrest in both G1 and G2 (Kastan *et al*, 1992). As shown in Figure 3I, these transcription factors were downregulated in Snai2-deficient MEFs in response to DNA damage by *γ*-irradiation. Thus, *γ*-irradiation is similar to doxorubicin in regulating the Snai2-dependent expression of these proteins. Moreover, the activation of Puma/bbc3 in both control and Snai2-deficient cells in response to *γ*-ray treatment was similar (Figure 3I), further indicating that Puma/bbc3 regulation in response to DNA damage is not affected in Snai2-deficient MEF.

### Effect of Snai2 on p53 target genes in cells undergoing growth arrest in response to DNA damage

**Figure 4:**
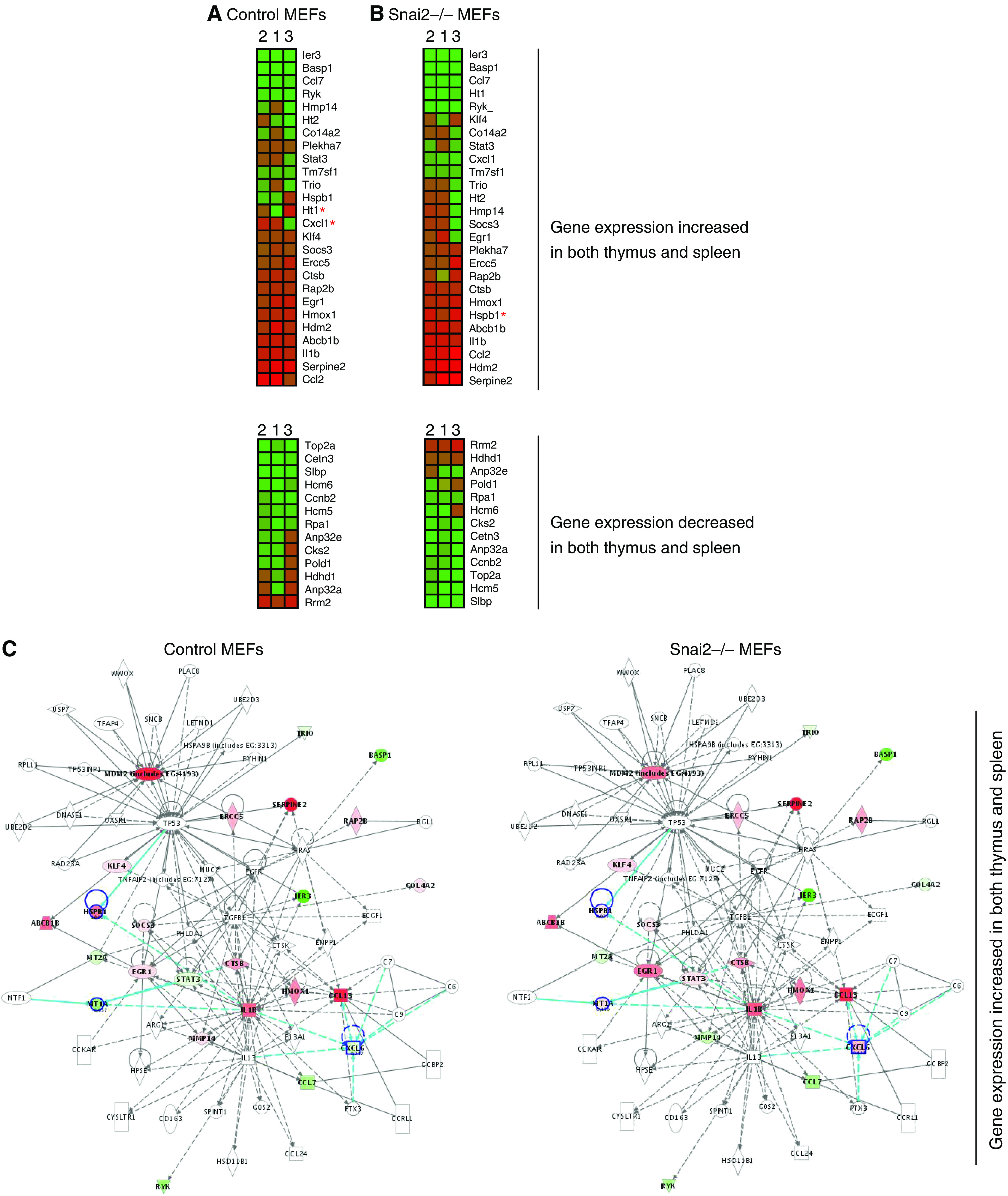
Graphical expression pattern in wild-type mouse embryonic fibroblasts (MEFs) (**A**) and Snai2-deficient MEFs (**B**) of genes upregulated and downregulated by p53 in response to DNA damage. Described genes upregulated and downregulated by p53 in the thymus and spleen (Burns and El-Deiry, 2003) in response to DNA damage were analysed. Each gene (identified at right) represented by a single row of coloured boxes; each independent experiment is represented by one single column. A red asterisk indicates major changes between control and Snai2-deficient MEFs. (**C**) Identification of Slug-dependent proteins using the Ingenuity database. Nodes are colour-coded in red (upregulated) or green (downregulated) according to their fold changes value.

**Figure 5:**
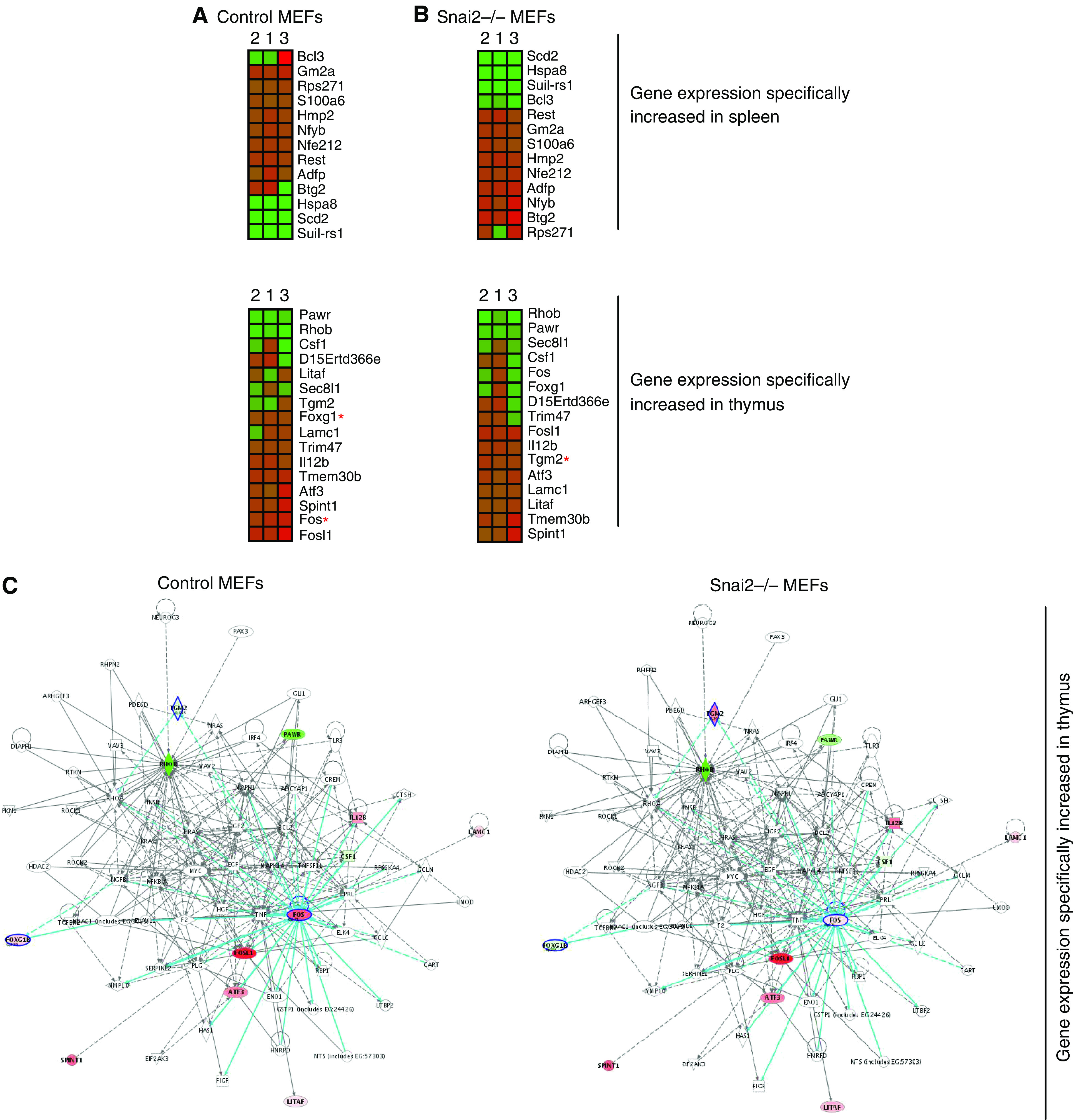

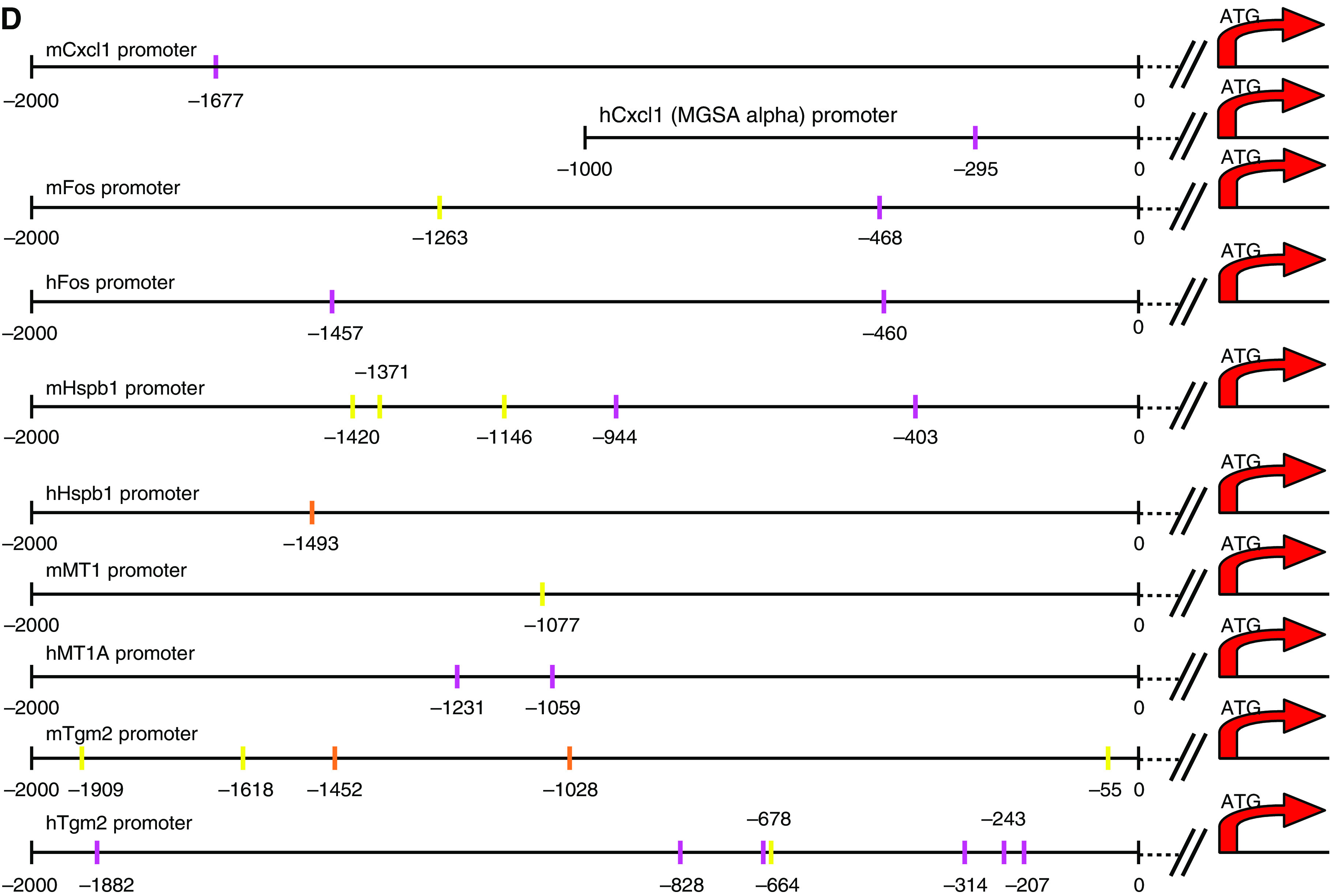
Graphical representation of the expression pattern in wild-type mouse embryonic fibroblasts (MEFs) (**A**) and Snai2-deficient MEFs (**B**) of genes known to be specifically increased by p53 in response to DNA damage. Genes known to be specifically increased by p53 in either thymus and spleen (Burns and El-Deiry, 2003) in response to DNA damage were studied. Each gene (identified at right) represented by a single row of coloured boxes; each independent experiment is represented by one single column. A red asterisk indicates the main expression differences between control and Snai2-deficient MEFs. (**C**) Identification of Slug-dependent proteins using the Ingenuity database. Nodes are colour-coded in red (upregulated) or green (downregulated) according to their fold changes value. (**D**) Identification of Snai2 DNA-binding sites within promoter regions of human and mouse of p53 target genes modulated by Snai2.

It is well established that there is a tissue specificity in the relative induction of several known p53 target genes in response to DNA damage (Burns *et al*, 2001; Burns and El-Deiry, 2003). Thus, we next examined how these genes were modulated in control and Snai2-deficient MEFs after doxorubicin treatment. As shown in Figures 4, 5 the overall expression of these p53 target gene expression patterns was similarly altered in both control and Snai2-deficient cells, indicating that p53 does not require Snai2 for regulation of majority of these genes in response to DNA damage in MEFs. However, the regulation of a significant number of p53 target genes in response to DNA damage in both control and Snai2-deficient MEFs differs from how they are regulated in the spleen and thymus in response to DNA damage (Burns *et al*, 2001; Burns and El-Deiry, 2003), confirming the striking cell specificity for p53-dependent transcription and repression (Burns *et al*, 2001; Burns and El-Deiry, 2003).

Analysis of the expression microarray data revealed six candidate p53 target genes (two genes whose expression was increased in Snai2-deficient MEFs compared to control MEFs in response to DNA damage: *Hspb1* and *Tgm2*; and four genes whose expression was decreased in Snai2-deficient MEFs compared to control MEFs in response to DNA damage: *Mt1*, *Cxcl1*, *Foxg1* and *Fos*). The Ingenuity program confirmed the existence in the Snai2-dependent transcriptome of six genes whose expression was altered in Snai2-deficient cells in response to DNA damage: *Hspb1*, *Tgm2*, *Mt1*, *Cxcl1*, *Foxg1* and *Fos* (Figures 4C and 5C). Good concordance was found between array and qPCR data for the selected p53 target genes regulated by Snai2 (Figure 3E), suggesting an interesting link between these genes and Snai2. These p53 target genes modulated by Snai2 belong mainly to the following categories on the basis of the biological or pathological function: metastasis (*Cxcl1*); cell cycle/DNA/oxidative damage (*Hspb1*, *Mt1*, *Fos*); survival (*Tgm2*), and cell development (*Foxg1b*). These results indicate that Snai2 regulates a limited set of p53 target genes in MEFs in response to DNA damage. Moreover, the presence of Snai2 DNA-binding sites (Inukai *et al*, 1999) in the promoter regions of these p53 target genes modulated by Snai2 (Figure 5D) suggests Snai2 could be directly involved in the control of transcription-repression of these targets.

## DISCUSSION

In this study, we have applied a functional genomics approach that combined gene expression profiling and computational molecular network analysis to obtain global dissection of the Snai2-dependent transcriptional response to DNA damage and to dissect the contribution of Snai2 on p53 target genes. As a model system, we used primary MEFs. Mouse embryonic fibroblasts represent an ideal cell system in which the activities of p53 can be studied. When treated with DNA-damaging agents, wild-type MEFs activate the cell cycle checkpoint by arresting in G1 (Attardi *et al*, 2000). This response is clearly p53 dependent as p53-null MEFs fail to undergo G1 arrest upon DNA damage treatment. Majority of approaches used to identify the role of p53-responsive genes such as *p21* have typically entailed comparing gene-expression profiles of cell lines lacking p53 with cell lines overexpressing p53 (El-Deiry *et al*, 1993; Okamoto and Beach, 1994; Buckbinder *et al*, 1995; Polyak *et al*, 1996). Another advantage of the strategy used here is that it relies on the response of endogenous cellular p53 to DNA damage and is performed using primary cells, making it very physiological.

Before using this approach to identify Snai2 target genes linked to *Snai2* gene function in response to DNA damage, we demonstrated that normal G1 arrest and normal p53 activation took place in Snai2-deficient MEFs in response to doxorubicin treatment. These observations indicated that p53 does not require Snai2 to activate the cell cycle checkpoint by arresting in G1. Moreover, these results indicate Snai2 does not require p53 for its DNA damage protective function in MEFs in agreement with previous data in haematopoietic precursors (Perez-Losada *et al*, 2002; Perez-Mancera *et al*, 2005; Wu *et al*, 2005). To confirm that our approach could be used to study the role of the p53-responsive gene *Snai2* in DNA damage response, we next examined whether known target genes were modulated in control and Snai2-deficient MEFs after DNA doxorubicin treatment. Expression of these genes was modulated as expected in control MEFs and their expression was not altered in Snai2-deficient cells in response to doxorubicin treatment. These results further confirm the approach used is physiologically relevant.

In addition to the elucidation of the genetic program of Snai2 and the identification of transcriptionally regulated protein networks, one of the main results of this work is the observation that p53 triggers similar transcriptomal programs in Snai2-deficient cells in response to DNA damage. However, these results do not rule out that the functional specificity of Snai2 may be established outside the transcriptional program or, alternatively, that it may require additional regulatory components and/or specific cell backgrounds. Further work in this area will be required to address these possibilities.

Another important observation of this work is the identification of a limited set of protein network (*Atm*, *Bid* and *p27*) and p53-target genes (*Hspb1*, *Tgm2*, *Mt1*, *Cxcl1*, *Foxg1* and *Fos*) whose expression is regulated by Snai2 in response to DNA damage. The presence of Snai2 DNA-binding sites (Inukai *et al*, 1999) in the promoter regions of these target genes modulated by Snai2 suggests Snai2 could be directly involved in the control of transcription-repression of these targets. Two of these targets (Atm and Bid) had already been identified as Snai2 targets describing the resistance to doxorubicin treatment of Snai2-expressing MCF7 cells (Kajita *et al*, 2004). In addition, Snai2 was shown in that study to bind Bid promoter (Kajita *et al*, 2004). The zinc-finger protein Snai2 is considered a transcriptional repressor. In agreement with this idea, expression of *Hspb1* and *Tgm2* was increased in Snai2-deficient MEFs compared to control MEFs in response to DNA damage. However, majority of Snai2-target genes (*Atm*, *Bid*, *p27*, *Mt1*, *Cxcl1*, *Foxg1* and *Fos*) were downregulated in Snai2-deficient MEFs in response to DNA damage, supporting the view that Snai2 can also behave as a positive transcriptional regulator or act by repressing the transcription of a repressor (Bermejo-Rodriguez *et al*, 2006). All these novel Snai2 targets have been implicated in DNA damage and survival regulation (Wang *et al*, 1991; Beattie *et al*, 1998; Park *et al*, 2000; Nanda *et al*, 2001; Katoh and Katoh, 2004; Minn *et al*, 2005). Thus, the regulation of these genes by Snai2 in response to DNA damage could be important in preserving integrity of tumour target cells and supports the view that failure to control Snai2 expression can produce cancer and alterations in development (Perez-Mancera *et al*, 2005; Perez-Mancera *et al*, 2006). These Snai2-target genes could represent novel pharmacological targets and/or biomarkers in cancers linked to Snai2 (Perez-Mancera *et al*, 2005; Bermejo-Rodriguez *et al*, 2006; Perez-Caro and Sanchez-Garcia, 2006).

The expression of the previously implicated Snai2-target gene Puma/bbc3 (Wu *et al*, 2005; Bermejo-Rodriguez *et al*, 2006) was not significantly modulated in Snai2-deficient cells in response to DNA damage, indicating that Puma/bbc3 regulation in response to DNA damage is not affected in Snai2-deficient MEFs. These results suggest that p53 does not require Snai2 for Puma/bbc3 regulation in response to DNA damage in MEFs, suggesting cellular context is of great importance for interpretation of Snai2 function. This tissue specificity of Snai2 is also supported by the fact that many Snai2-expressing lineages showed no obvious phenotypes in Snai2 mutant mice (Jiang *et al*, 1998; Perez-Losada *et al*, 2002; Sanchez-Martin *et al*, 2002, 2004), suggesting that Snai2 function in these cell types either is not required or can be compensated through synergy with other Snail family members. Moreover, this observation is in agreement with the known tissue specificity in the relative induction of several known p53 target genes (Burns *et al*, 2001; Burns and El-Deiry, 2003). Our results clearly show that the regulation of a significant number of p53 target genes in response to DNA damage in both control and Snai2-deficient MEFs differ from how they are regulated in the spleen and thymus in response to DNA damage (Burns and El-Deiry, 2003), confirming the striking cell specificity for p53-dependent transcription and repression.

In summary, these results have provided a comprehensive picture of the transcriptional events regulated by Snai2 during the DNA-damage process. From this analysis, we have obtained information regarding the Snai2-dependent molecular routes induced by DNA damage, defined the effect of Snai2 on p53 target genes, pointed to novel putative Snai2 targets, and suggested a mechanistic model. The emerging model suggests that targeting the Snai2-mediated arm could effectively increase the radiosensitivity of Snai2-dependent cancers. Given that our work has been focused on MEF cells, it would be interesting to expand these studies to other cell types in the future to get an idea of the level of conservation of the transcriptional programs of Snai2 in different tissues. Future studies will define if cancer maintaining cells (or cancer stem cells) keep constitutively active part of the original genetic programme controlled by Snai2 in MEFs in response to DNA damage.
